# Laws against Quackery

**Published:** 1887-01-01

**Authors:** 


### LAWS AGAINST QUACKERY.

Upon this subject Dr. Becton, of the Texas Medical Association, in his annual address before that body, remarked that "the educated physician needs no protection except such as the law gives every good citizen. Quacks cannot be suppressed by legal enactments. They find place and favor in all professions and avocations, and are simply the outgrowth of human corruption, and doubtless will be found in all human institutions this side of the millenium. Vile imposters and pretenders are found in the pulpit, at the bar, in politics—everywhere ; and every effort to rid our profession of them and to elevate the standard of honorable medicine by legislative enactments will prove abortive." He remarked further upon the fact that the people, who need protection, do not desire it, and do not ask for it ; and he might have added that the people have uniformly opposed the demands of the profession in this direction. The masses are with the Quacks, and there appears to be an antagonism to this kind of legislation in our constitutional system, and in the customs, instincts and historical traditions of the people, which nought but Time and enlightened civilization can eradicate.

While we differ with Dr. Becton, in part, we are constrained to admit that there are grave difficulties in securing to legitimate medicine that protection that seems to be demanded. The protection required is not so much to the profession as to the people, who, in their ignorance of medicine, are made the dupes of mountebanks of every kind. The profession should not so much seek protection for themselves as for the people. The honorable physician must rise superior to all selfish aims and purposes in the matter of seeking legislation against Quackery. The Regular profession would be in much better condition to accomplish good in this direction if they could but purge their ranks of the many impostors who, while proclaiming so often and loudly for the Ethics, are secretly availing themselves of all the tricks and methods of the worst forms of Empiricism.

In the Georgia Medical Association it is contemplated, at its next meeting, to discuss the propriety of adopting, in Georgia, the Alabama method of organizing the profession. In this plan, the Legislature of the State is expected to unite with the Association in protecting the people against incompetent medical men, and in certain regulatious relating to Health Boards, etc. We are told the law has worked well in Alabama, and yet there is an amount of machinery and detail about it that we fear would be very difficult to carry out. We have not yet studied its details with suffieient care, and are scarcely prepared now to speak advisedly upon this important subject.

We learn that the Alabama law is now meeting with great opposition in that State by the Eclectics, who have organized against it, and are memorializing the Legislature against it, as bearing unequally and as violative of constitutional rights, etc. In the opposition they find, as usual in all such cases, numerous allies in the legal profession and among the people at large, and many Buncombe legislators are ready to inveigh against what they call a partial and unjust law. So it has ever been, and so it will probably be in Georgia should we, after a long struggle, succeed in securing the required legislation to carry out fully the Alabama method.

